# The prevalence of tuberculosis among prisoners in Ethiopia: a systematic review and meta-analysis of published studies

**DOI:** 10.1186/s13690-017-0204-x

**Published:** 2017-08-21

**Authors:** Addisu Melese, Habtamu Demelash

**Affiliations:** 1 Department of Medical Laboratory Science, College of Health Sciences, Debre Tabor University, Debre Tabor, Ethiopia; 2 Department of Public Health, College of Health Sciences, Debre Tabor University, Debre Tabor, Ethiopia

**Keywords:** Tuberculosis, Prison, Prisoner, Systematic review, Meta-analysis, Ethiopia

## Abstract

**Background:**

Except individual studies with varying prevalence rates, there are no national prevalence studies conducted in prison settings in Ethiopia. Appropriate estimates of the disease is essential to formulate health service plans most fitted for prisoners. Therefore, this systematic review and meta-analysis was designed to pool the results of individual studies and estimate the prevalence of tuberculosis among prisoners in Ethiopia.

**Methods:**

MEDLINE/PubMed, Cochran library, and Google scholar databases were searched for potential studies on the prevalence of tuberculosis among prisoners in Ethiopia. A total of 177 titles were identified and 10 studies met the inclusion criteria. Descriptive and quantitative data of the included studies were presented in tables and forest plots. Potential sources of heterogeneity across studies were assessed using the Cochrane’s Q and I^2^ tests. The MetaXL (version 5.3) was employed to compute the pooled prevalence of TB using the random effect model and 95% confidence interval.

**Result:**

Based on the ten studies included in the meta-analysis, about 4086 prisoners were infected with tuberculosis (TB). The pooled prevalence of TB among prisoners was therefore 8.33% (95% CI; 6.28%–10.63%) and the pooled point prevalence was estimated at 888 per 100,000 prison population (95% CI; 531–1333). The prevalence of TB using microscopy alone was 6.59% (95% CI: 3.96–9.50%) whereas the prevalence of TB when microscopy is combined with either culture or molecular tests was 8.57% (95% CI: 4.94–12.6%).

**Conclusion:**

The pooled prevalence of tuberculosis among prisoners in Ethiopia is expectedly high. This high prevalence could explain the spread of TB within prisons and between prisoners and varies communities. Thus; attention should be given to prison settings to prevent the transmission and emergence of drug resistance TB both in inmates and general population. Further studies covering large scale prison population are needed to design effective diagnostic, treatment and preventive methods.

**Electronic supplementary material:**

The online version of this article (doi:10.1186/s13690-017-0204-x) contains supplementary material, which is available to authorized users.

## Background

Globally, the prevalence of tuberculosis (TB) among prisoners is greater than the general population [1]. Although the burden is well noticed, prisons are often overlooked by national health sectors and are not included in the national health statistics [2]. Living in congregate settings where both TB patients and uninfected inmates frequently crowded increases the risks of contracting infection and developing multi-drug resistant TB [3, 4].

Although prisons have healthcare centers that provide diagnosis and treatment of TB for both prisoners and prison staffs in Ethiopia, the services are provided through referral systems to outside healthcare centers [5–8]. The infection control practices in the receiving healthcare centers is usually poor. A study conducted on the practice of TB infection control among healthcare workers in Ethiopia showed that only 38% of them practiced the control plans properly [9]. Laboratories are often inadequate and delays both screening and referral systems [10]. These all worsens the rate of transmission and become important reservoir of TB infection for inmates and general population.

Different factors might be attributable for the higher prevalence of TB in prisons. A study conducted in eastern Ethiopian prisoners showed that the knowledge of prisoners on the cause of TB was poor. Only 1.6% of the prisoners knew the causes of TB [11]. Other studies conducted in prisoners in Ethiopia reported that the risks of developing tuberculosis were associated with undernutrition [12, 13], illiteracy [14], smoking [12, 13, 15], increased duration of imprisonment [6, 13], overcrowding and poor ventilation [13, 16], reproductive age (15–44 years) and urban residence before imprisonment [17], contact history with TB patients [8, 12, 14, 17, 18] and previous TB infection [15].

Systematic screening of contacts and high-risk groups is one of the pillars of the global End TB strategy [19]; but the health services of prisons are often overlooked and underfunded creating opportunities for prisons to receive, concentrate and disseminate TB within and to outside population [2, 20]. Despite the integration of TB care in prisons in the national TB prevention and control programs of Ethiopia, there is no systematic screening of detainees on admission for potential infectiousness to prevent transmission, disability and death [4]. Therefore, prisoners infected and contagious for TB might be added to crowded cells and became sources of infection [20].

Results from Ethiopian prisons showed a varying prevalence rates; ranging from 1.8 to 19.4% [7, 15] and diagnoses were highly dependent on microscopy. Nevertheless, through strong screening and use of sensitive laboratory tests, the prevalence could be potentially higher than the reported one. Lack of national summarized data could be one of the reasons for the poor implementation of TB prevention and control programs in prisons. Except individual studies with varying prevalence rates, there are no national prevalence studies conducted in prison settings in Ethiopia. Appropriate estimates of the disease is essential to formulate health service plans most fitted for prisons settings. Therefore, this systematic review and meta-analysis was done to estimate the pooled prevalence of TB for better understanding of the burden of TB in Ethiopian prisons.

## Methods

### Data sources and search strategy

MEDLINE/PubMed, Cochrane library and Google scholar databases were systematically searched for studies reporting the prevalence of TB among prisoners in Ethiopia following the Preferred Reporting Items for Systematic Reviews and Meta-Analyses (PRISMA) statement guideline [21]. The electronic search was done for the MEDLINE/PubMed database using the following Medical Science Heading (MeSH) terms: (“tuberculosis”[MeSH Terms] OR “tuberculosis”[All Fields]) OR TB[All Fields] OR MTB[All Fields] OR (“mycobacterium tuberculosis”[MeSH Terms] OR (“mycobacterium”[All Fields] AND “tuberculosis”[All Fields]) OR “mycobacterium tuberculosis”[All Fields]) AND (“prisons”[MeSH Terms] OR “prisons”[All Fields] OR “prison”[All Fields]) OR (“prisoners”[MeSH Terms] OR “prisoners”[All Fields]) OR (“prisoners”[MeSH Terms] OR “prisoners”[All Fields] OR (“prison”[All Fields] AND “inmates”[All Fields]) OR “prison inmates”[All Fields]) AND (“ethiopia”[MeSH Terms] OR “ethiopia”[All Fields]). The search was limited to the study category of humans and English language publications. The literature search was limited to studies published up to October 30, 2016 and the bibliographies of relevant articles were also hand searched.

### Box 1: Eligibility criteria

**Table:** 

Inclusion criteria
• Country and setting: Ethiopian prisons • Study design: Cross-sectional • Reported the prevalence or number of TB cases • Peer-reviewed and published in English language • Undertook laboratory works and reported type of laboratory tests • Reported type of specimen used for laboratory works • Reported type of TB identified • Reported quality control/assurance measures
Exclusion criteria
• Reported the knowledge and practice of prisoners towards TB disease • Investigated patterns of drug resistance only • Reviews that reiterated findings from the already included studies

### Study selection, quality assessment and data extraction

Among the articles identified, titles and abstracts were reviewed to retrieve studies on the prevalence of TB. Articles found relevant by title and abstract were undergone for full text review for eligibility. The quality of eligible studies was assessed against predefined inclusion criteria and the Health states Quality scale using the quality effects model [23]. Studies having 50% and above quality score were included in the analysis (Additional file 1). Data were extracted using Microsoft Excel and includes: author & year of publication, region, year of survey, study design, sample size, inclusion criteria, type of specimen, diagnostic method used and type of TB identified, number of TB cases and prevalence rates. The study selection, quality assessment and data extraction was done from August 1 to December 30, 2016. Two reviewers (AM, HD) conducted the selection and quality assessment of articles independently. Inconsistencies between the reviewers was solved by discussion and articles were included after consensus was reached.

### Definitions

- Tuberculosis is an infectious disease caused by the bacillus *Mycobacterium tuberculosis* that typically affects the lungs (pulmonary TB) and spread when people who are sick with pulmonary TB expel bacteria into the air but can also affect other sites (extrapulmonary TB) [19].
- Prisoner in this study is used to describe anyone held in prisons during investigation of a crime, anyone awaiting a trial, and anyone who has been sentenced.
- The primary outcome measure was the percentage of individuals having TB disease. The prevalence of TB was calculated by dividing the number of prisoners with TB disease by the total number of study subjects, and multiply by 100.
- The point prevalence per 100,000 prisoners was calculated by dividing the number of prisoners with TB disease by total number of prisoners and multiplied by 100,000.

### Data analysis

The extracted data were entered and analyzed using MetaXL version 5.3 software. Potential sources of heterogeneity across studies was evaluated by Cochrane’s Q test (which verifies the presence of heterogeneity) and I^2^ statistics (which shows the amount of heterogeneity between studies). The I^2^ provides the percentage of variability due to heterogeneity rather than chance difference or sampling error. The I^2^ > 50% and Q test (*P* < 0.10) was considered statistically significant heterogeneity. The random effects model (DerSimonian-Laird method) [22] which assesses the variability within and between studies was applied to estimate the pooled prevalence and 95% CIs. In order to address the problems of confidence limits and variance instability that could arise from single studies with small or larger prevalence rates, the transformed double arcsine method was employed.

Publication bias was assessed using the Doi plot and Luis Furuya-Kanamori asymmetry index (LFK index). In the presence of symmetry, one can concluded as no publication bias but in the absence of symmetry, one can expect publication bias. This publication bias was measured by asymmetry index (LFK index). An LFK index within ±1, out of ±1 but within ±2, and > ± 2 is to mean no asymmetry, minor asymmetry and major asymmetry, respectively [23]. Sensitivity test was done to give a quick indication which study is the prime determinant of the pooled result, and which is the main source of heterogeneity. The test excludes each study one by one in the analysis to show the pooled effect sizes and associated heterogeneity. Subgroup analysis was done by type of diagnostic methods used during the survey of each studies.

## Results

### Characteristics of included studies

**Fig. 1:**
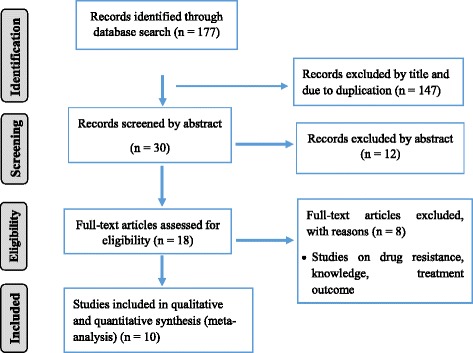
PRISMA flow chart of study selection

The search strategy retrieved 177 potential articles, of which 18 were screened as full text articles and ten studies comprising of 4086 prisoners were found eligible and included in the analysis (Fig. 1). Among the articles included in the analysis, four studies [5–7, 13] were conducted on a single prison facility and six studies [8, 12, 14, 15, 17, 18] were conducted on two or more than two prison facilities. All were cross-sectional studies with a study population ranging from 124 prisoners in Gamo Gofa [13] to 1223 in Tigray [8] and conducted from 2011 to 2016.

The studies were conducted in different regions of the country; Amhara [5, 6, 12, 14], Tigray [8] and Southern Nations, Nationalities and Peoples (SNNP) [7, 13, 15]. Two studies were conducted across regions [16, 18]. Both the lowest and highest prevalence rates were reported from the SNNP region; Hadiya and Wolaita, respectively [7, 13]. Five studies employed microscopy (either Ziehl-Neelsen or light emitting diode) for bacteriological confirmation of TB [5–7, 12, 17] and the remaining studies combined microscopy with either culture or molecular tests [8, 13–15, 18]. None of the studies reported chest X-ray as a screening or diagnostic tool; that all studies used questionnaires on symptoms of TB followed by diagnostic testing of prisoners suspected of having TB infection. Nine (90%) of the included studies reported the mean numbers of prisoners per cell and were ranging from 64 [8] to 333 [5] prisoners. Six studies reported mean length of imprisonment and ranged from 7.5 [17] months to 3 years [5]. Four studies provided information on HIV status and the TB-HIV coinfection ranged from 4.23% [18] to 34.6% [5]. Only one study [12] assessed the presence of drug resistant TB cases using GeneXpert MTB/RIF assay. Study characteristics are summarized in Table 1.

**Table 1:** Summary of studies assessing the prevalence of tuberculosis among prisoners and included in the analysis

Author, ref	Study region	Study design	Study period	Inclusion criteria	Sample size	Diagnostic method used	Specimen	Type of TB identified	No of cases, N (%)	Point prevalence per 100,000 pop
Moges et al., 2012 [5]	Amhara	Cross-sectional	March to May 2011	Cough ≥1 week	250	LED microscopy, cytology	Sputum, FNAC	SPPTB	26 (10.4)	1482.3
Abebe et al., 2011 [17]	Dire Dawa, Somali, Harari	Cross-sectional	July to November 2008	Cough ≥2 week, on anti-TB treatment	382	ZN microscopy, culture	Sputum	PTB	44 (11.5)	1913
Addis et al., 2015 [6]	Amhara	Cross-sectional	February to July 2008	Cough ≥2 week, on anti-TB treatment	384	ZN microscopy	Sputum	SPPTB	33 (8.59)	2032
Bayu et al., 2016 [13]	SNNP	Cross-sectional	March to April 2015	Cough ≥2 week, on anti-TB treatment	305^b^	ZN microscopy	Sputum	PTB	17^b^ (5.57)	966
Fuge et al., 2016 [7]	SNNP	Cross-sectional	May to June 2013	Cough ≥1 week, > = 15 years	164	ZN microscopy	Sputum	SPPTB	3 (1.83)	349.2
Zerihun et al., 2015 [15]	SNNP	Cross-sectional	Nov. 2011 to March 2012	Cough ≥2 weeks	124	ZN microscopy, culture	Sputum	PTB	24 (19.35)	629
Biadglegne et al., 2014 [14]	Amhara	Cross-sectional	November 2013	Cough ≥1 week, sputum production	207^b^	ZN microscopy, culture, GeneXpert	Sputum	SNPTB	23^b^ (11.1)	-
Ali et al., 2015 [18]	Oromia, SNNP, Harari	Cross-sectional	January to December 2013	≥18 years and either HIV+, treatment in the last 5 years, or WHO grade 5 TB identification criteria^a^	765	ZN microscopy, culture	Sputum	PTB	71 (9.3)	458.2
Gebrecherkos et al., 2016 [12]	Amhara	Cross-sectional	February to April 2015	Cough ≥2 weeks, not on anti-TB treatment	282	ZN and LED microscopy, GeneXpert	Sputum	SPPTB	15 (5.3)	384.6
Adane et al., 2015 [8]	Tigray	Cross-sectional	August 2013 to February 2014	≥ 18 years, not on anti-TB treatment, WHO grade 5 TB identification criteria^a^	1223^b^	ZN, culture	Sputum	PTB	74^b^ (5.88)	793.5

*SNNP* Southern nations, nationalities and peoples, *TB* Tuberculosis, *SPPTB* Smear positive pulmonary tuberculosis, *PTB* pulmonary tuberculosis, *SNPTB* Smear negative pulmonary tuberculosis, *ZN* Ziehl-Neelsen, *LED* Light emitting diode, *FNAC* Fine needle aspiration cytology

^a^WHO grade 5 TB identification criteria: cough > = 2 weeks, sputum production, chest pain, loss of appetite, weight loss in last 3 month

^b^The number of on anti-TB treatment prisoners were added to the sample size as well as to the reported numbers of cases to estimate the prevalence

### Heterogeneity and publication bias

**Fig. 2:**
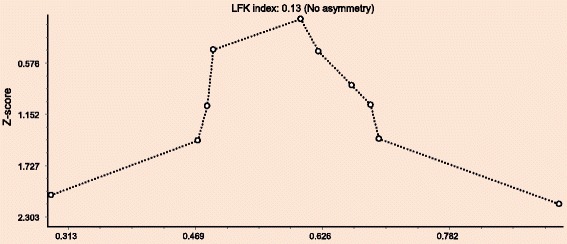
Doi plot analysis and LFK index of publication bias

The included ten studies were assessed for heterogeneity and publication bias. Accordingly, the analysis showed a substantial heterogeneity of Q test (*p* < 0.001) and I^2^ statistics (I^2^ = 83%). The Doi plot for publication bias showed no symmetry verifying the presence of bias but no evidence of bias by the asymmetry index (LF index = 0.13) (Fig. 2).

### Sensitivity analysis

Sensitivity analysis of the ten studies was done to test the effect of each study on the pooled result by excluding each study step by step (i.e based on nine studies) and the results showed that two studies (Fuge et al., 2016 & Zerihun et al., 2015) were relatively the prime determinants of the pooled result while relatively higher source of heterogeneity comes from the study Addis et al., 2015 [6] (Table 2).

**Table 2:** Summary of sensitivity analysis of the included studies

Excluded studies	Pooled prevalence (95% CI)	I^2^ (95% CI)	*P*-value
Moges et al., 2012	8.10 (5.93, 10.57)	83.99 (72.23, 91.09)	<0.001
Abebe et al., 2011	7.97 (5.86, 10.36)	82.44 (67.98, 90.37)	<0.001
Addis et al., 2015	8.29 (6.01, 10.89)	84.57 (72.43, 91.37)	<0.001
Bayu et al., 2016	8.65 (6.40, 11.20)	83.91 (71.06, 90.06)	<0.001
Fuge et al., 2016	9.06 (7.09, 11.29)	79.42 (61.50, 89.00)	<0.001
Zerihun et al., 2015	7.61 (5.83, 9.60)	78.23 (58.88, 88.47)	<0.001
Biadglegne et al., 2014	8.05 (5.92, 10.47)	83.77 (70.77, 90.99)	<0.001
Ali et al., 2015	8.21 (5.87, 10.89)	83.93 (71.10, 91.06)	<0.001
Gebrecherkos, 2016	8.68 (6.44, 11.21)	83.77 (70.76, 90.99)	<0.001
Adane, 2015	8.66 (6.32, 11.31)	81.48 (65.94, 89.93)	<0.001

### Pooled prevalence of TB among prisoners

**Fig. 3:**
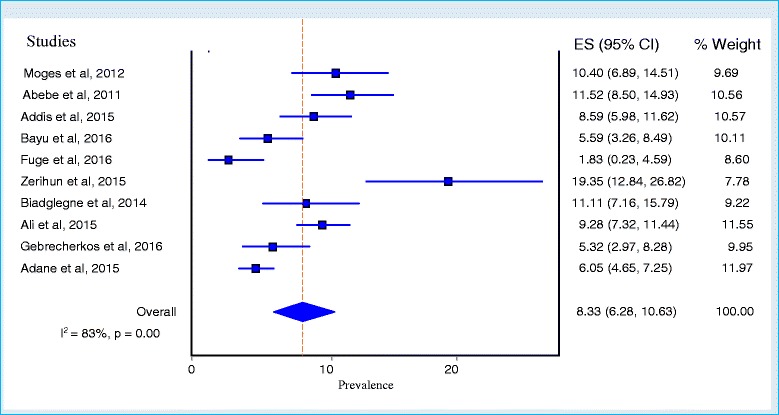
Forest plot of the pooled prevalence of tuberculosis among prisoners in Ethiopia

**Fig. 4:**
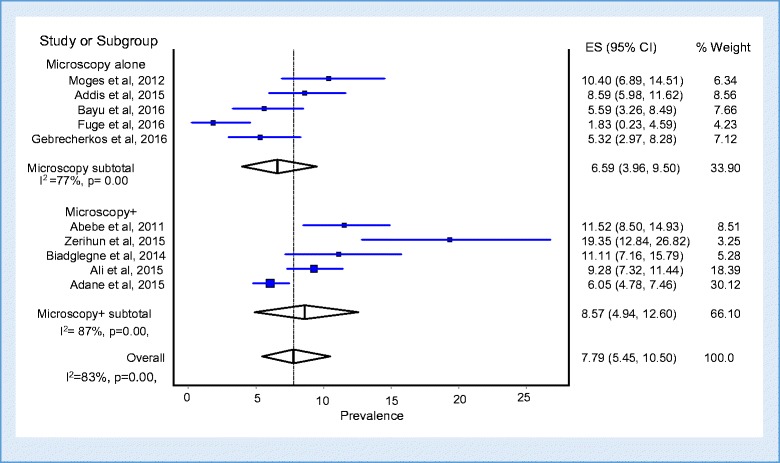
Forest plot of the pooled prevalence of tuberculosis by type of diagnostic methods used during the survey using quality effects model

The prevalence estimates of TB among prisoners is presented in a forest plot (Fig. 3). The prevalence of each study ranged from 1.83 to 19.35% with a substantial heterogeneity across studies (Q = 53.69; *p* < 0.001; I^2^ = 83%; 95% CI = 69.65–90.20). The overall pooled prevalence of TB from the random effects method revealed a prevalence of 8.33% (95% CI; 6.28–10.63, *p* < 0.001). The pooled point prevalence from the nine studies that reported prevalence rates per 100,000 prison population was estimated to be 888 per 100,000 prison population (95% CI; 531–1333). In the Subgroup analysis for the prevalence of TB by type of diagnostic methods showed that the prevalence of TB when diagnosed by microscopy alone was 6.59% (95% CI; 3.96–9.50%) and when microscopy was combined with either culture or molecular tests was 8.57% (95% CI; 4.94–12.6%) (*p* < 0.001) (Fig. 4).

## Discussion

The majority of studies included in this analysis reported a high TB prevalence rates in prisoners in Ethiopia. Although the ten studies contained information on the prevalence of TB, none of the studies provided information on the prevalence of latent TB in prisoners. The included studies used various inclusion criteria, diagnostic assays and sampled only suspected cases or did not include on treatment patients. Most of the studies were conducted in the Amhara and SNNP regions with no information about the burden in the capital (Addis Ababa), Afar, Gambella and Benishangul Gumuz regions.

TB in prisons is often overlooked and continued to be a major public health problem in many settings, particularly in countries with a high incidence of TB, including Ethiopia [2]. To the best of our information, this is the first meta-analysis conducted in Ethiopia to determine the pooled prevalence of TB among prisoners. Accordingly, the pooled prevalence estimate of TB among prisoners in Ethiopia was 8.33% (95% CI; 6.28, 10.63, *p* < 0.001). This pooled prevalence is comparable with reports from South Africa prison with a prevalence rate of 8.8% [24].

The pooled prevalence result, on the other hand, was less than prevalence reports from Zambian prisons (22.7%) [25] and Democratic Republic of Congo (17.7%) [26]. This might be due to the laboratory tests used to diagnose TB. In Ethiopia, TB diagnosis relies on microscopy which has low detection rate and 50% of the included studies used microscopy as a confirmation of TB. In addition, screening was solely dependent on symptoms of cough and none of the included studies used chest X-ray as a screening and diagnosis of TB. Moreover, screening during admissions to prisons was not done for which it could increase the prevalence by participating larger numbers of prisoners. A study from Tigray [8] also reported that half of pulmonary TB cases were left undiagnosed in prisons which supports the evidence of poor case detection rate in prisons.

Our findings are relatively higher than reports from similar settings in Brazil (4.5%) [27], Malawi (1.4%, 5.1%) [28, 29] and Cameroon (3.3%) [30]. A previous series report on HIV and related infections in prisoners also reported a pooled prevalence of 5.3% in Eastern and Southern Africa (where Ethiopia is located) [31], relatively lower than our finding. The relative higher pooled prevalence in our study could be attributed for the small size of prison population participated in the study which is pooled using only 4086 prisoners from the ten studies.

The pooled point prevalence was also estimated from nine studies. From the analysis, the point prevalence was found to be 888 per 100,000 (95% CI; 531–1333). Although in agreement with reports from Brazil prisons with a point prevalence of 917/100,000 [27], it was three times higher than the point prevalence of the general population in Ethiopia (277/100,000) [32]. Recent reports showed a national prevalence of 200/100,000 [33], four times lower than our finding. On the other hand, lower prevalences were reported from prison settings; 696/100,000 in Malawi [28], 341/100,000 in Turkey [34] and 215/100,000 in France [35]. The lower prevalences in these countries could be attributed for strong TB control strategies, low incidence rates and established good health systems both in prisons and general population. Other studies from prison settings in South Africa [24], Zambia [25], and Cameroon [36] reported much higher point prevalences; 8772, 4005 and 3197; respectively).

In addition, subgroup analysis was done from quality effects model to see the differences in prevalence rates by type of diagnostic tests used. Accordingly, significant difference was observed across diagnostic tests. The pooled prevalence of TB using microscopy alone was 6.59% (95% CI: 3.96–9.50; I^2^ = 77%; *p* < 0.001) while the pooled prevalence of TB using microscopy combined with culture or GeneXpert was 8.57% (95% CI: 4.94–12.60; I^2^ = 87%; *p* < 0.001). This is clearly known that microscopy combined with sensitive tests including culture and molecular tests improves the detection rate of TB.

In meta-analyzing prevalence studies, studies with smaller or larger prevalence reports could affect the pooled result by giving wider confidence intervals and variance instability. The sensitivity analysis of our study showed that two smaller studies (Fuge et al. 2016 & Zerihun et al. 2015) having smallest and largest prevalence rates, respectively, were the prime determinants of the pooled result. We, therefore, combined them with other studies in to a weighted average to minimize the variance; so that larger studies and studies with lesser variation could have greater weight and vice versa in the final combined estimate by using the random effects model (see % weight column in Fig. 3).

This study has certain limitations. Included studies were cross-sectional and limited in number (only ten studies) providing only snapshots of the situation at a particular moment in time and fail to capture the dynamic nature of the prison population. Diagnostic methods of TB were also varied between studies which could affect the pooled results. Moreover, lack of information and data from some regions including the capital Addis Ababa, made it difficult to generalize the findings. In addition, this study was based only on published peer-reviewed studies and important data might be missed from unpublished studies and grey publications.

## Conclusions

The pooled prevalence of tuberculosis among prisoners in Ethiopia is expectedly high. This high prevalence could explain the spread of TB within prisons and between prisoners and varies communities. Thus; attention should be given to prevent the transmission and emergence of drug resistance TB both in inmates and general population. Moreover, no evidence was found on the situation of latent TB and evidences on the effect of HIV on the occurrence of TB were conflicting. Further studies covering large scale prison population are needed to design effective diagnostic, treatment and preventive methods. Strengthening prison healthcare centers with manpower and infrastructure, screening during admission, periodic screening for TB symptoms and active case finding, trainings for prisoners and prison staffs on TB infection prevention, supporting diagnosis with CXR and culture and/or molecular tests, screening for latent infections and prompt treatments can be used as an immediate response to curve the burden and to make prisons not further be reservoirs.

## Additional file

Additional file 1: The Health states Quality scale and quality score. (DOCX 17 kb)

## Abbreviations

AFB: Acid fast bacilli
CXR: Chest X-ray
PTB: Pulmonary tuberculosis
SNPTB: Smear negative pulmonary tuberculosis
SPPTB: Smear positive pulmonary tuberculosis
TB: Tuberculosis
