# Supplementary material for: The prevalence of tuberculosis among prisoners in Ethiopia: a systematic review and meta-analysis of published studies
Source: Arch Public Health. 2017 Aug 21;75:37. doi: 10.1186/s13690-017-0204-x (PMC5563894; doi:10.1186/s13690-017-0204-x)
Supplement: Additional file 1: — The Health states Quality scale and quality score. (DOCX 17 kb) [file 13690_2017_204_MOESM1_ESM.docx]

**The Health states Quality scale**

| Studies | Quality scale variables | | | | | | Total  (max 12) | Score |
| --- | --- | --- | --- | --- | --- | --- | --- | --- |
|  | 1 | 2 | 3 | 4 | 5 | 6 |  |  |
| Moges et al, 2012 | 1 | 1 | 1 | 2 | 1 | 2 | 8 | 0.67 |
| Abebe et al, 2011 | 1 | 2 | 1 | 2 | 2 | 2 | 10 | 0.83 |
| Addis et al, 2015 | 1 | 1 | 1 | 2 | 1 | 2 | 8 | 0.67 |
| Bayu et al, 2016 | 1 | 1 | 2 | 2 | 1 | 2 | 9 | 0.75 |
| Fuge, 2016 | 1 | 1 | 1 | 2 | 1 | 2 | 8 | 0.67 |
| Zerihun, 2015 | 1 | 2 | 1 | 2 | 2 | 2 | 10 | 0.83 |
| Biadglegne, 2014 | 1 | 2 | 1 | 2 | 2 | 1 | 9 | 0.75 |
| Ali, 2015 | 1 | 2 | 1 | 2 | 2 | 2 | 10 | 0.83 |
| Gebrecherkos, 2016 | 1 | 1 | 1 | 2 | 2 | 2 | 9 | 0.75 |
| Adane, 2016 | 1 | 2 | 1 | 2 | 2 | 2 | 10 | 0.83 |

**The Health states Quality scale variables**

| 1. Were the target population and the study period clearly defined? 2. Yes = 1 3. No = 0 |
| --- |
| 1. Diagnostic methods and quality control 2. Use of sensitive tests (culture, GeneXpert) = 2 3. Use of microscopy alone = 1 4. Symptom based/not specified = 0 |
| 1. Method of selection 2. Attempts all cases = 3 3. Set criteria = 2 4. Convenience sampling/other = 1 5. Not specified = 0 |
| 1. Administration of measurement and quality control 2. Administered interview = 2 3. Register/case record = 1 4. Not specified = 0 |
| 1. Study Area 2. Broad (multi-site survey, > 2) = 2 3. Small (single site surevy) = 1 4. Not specified = 0 |
| 1. Outcome measurement 2. Both point and per 10^5^ prevalence = 2 3. Point prevalence alone = 1 4. Not specified = 0 |
